# Cross-frequency coupling between low frequency and gamma oscillations altered in cognitive biotype of depression

**DOI:** 10.3389/fpsyt.2025.1596191

**Published:** 2025-06-10

**Authors:** Hongli Wang, Xiaoning Shi, Chenyang Wang, Yongsheng Qi, Michel Gao, Yingying Zhao

**Affiliations:** ^1^ Beijing Key Laboratory of Mental Disorders, National Clinical Research Center for Mental Disorders & National Center for Mental Disorders, Beijing Anding Hospital, Capital Medical University, Beijing, China; ^2^ Advanced Innovation Center for Human Brain Protection & Laboratory for Clinical Medicine, Capital Medical University, Beijing, China; ^3^ WM Therapeutics Co. Ltd, Beijing, China

**Keywords:** depression, cognitive biotype, gamma oscillations, cross-frequency coupling, parietal lobes

## Abstract

**Aim:**

The cognitive biotype of depression has been conceptualized as a distinct subtype characterized by unique distinct neural correlates and specific clinical features. Abnormal neural oscillations related to cognitive dysfunction have been extensively studied, with particular attention given to gamma oscillations due to their crucial role in neurocircuit operations, emotional processing, and cognitive functions. Nevertheless, cross-frequency coupling between low frequency and gamma oscillations in the cognitive biotype of depression have yet to be fully elucidated.

**Method:**

The study identified the cognitive biotype in depression by MATRICS Consensus Cognitive Battery (MCCB). We enrolled 141 depressed patients in remission, including 56 identified as cognitive biotype and 85 as the non-cognitive impairment subgroup. Cross-frequency coupling between low frequency and gamma oscillations were analyzed using specific computational methods based on the data collected by Electroencephalogram (EEG). Furthermore, we did correlation analysis to explore the relationship between cross-frequency coupling of neural oscillations with cognitive function in depression.

**Results:**

We found that phase-amplitude coupling (PAC) values decreased in cognitive biotype. Specifically, cross-frequency coupling between theta (Pz: t =-3.512, FDR-corrected p = 0.011), alpha (P3: t =-3.377, FDR-corrected p = 0.009; Pz: t =-3.451, FDR-corrected p = 0.009), beta (P3: t =-3.129, FDR-corrected p = 0.020; Pz: t =-3.333, FDR-corrected p = 0.020) with low gamma decreased at eyes-closed state in cognitive biotype. However, cross-frequency coupling between delta with gamma increased in cognitive biotype (P4: *t* = 3.314, FDR-corrected p = 0.022) While cross-frequency coupling exhibited no significant differences at eyes-opened state in two subgroups (FDR-corrected p > 0.05). Furthermore, significant correlations between cognitive function and cross-frequency coupling at eyes-closed state were observed.

**Conclusion:**

These results indicated that the cross-frequency coupling between low frequency and gamma occurred in the parietal lobe in cognitive biotype of depression. These results advance the understanding of neurophysiological mechanisms underlying cognitive deficits and highlight potential biomarkers for precision depression.

## 1 Introduction

Depression is a heterogeneous psychiatric disorder with diverse clinical manifestations and underlying neurobiological mechanisms (1, 2). Recent advances in neurophysiological research have identified distinct biotypes of depression, with the cognitive biotype emerging as a particularly significant subtype characterized by prominent cognitive dysfunction and specific neural circuit abnormalities. This biotype is associated with impaired executive function, attentional deficits, and working memory disturbances, which significantly contribute to functional disability and poor treatment outcomes (3).

Neural oscillations, particularly in the gamma frequency range (30-90 Hz), have been implicated in the pathophysiology of depression due to their critical role in cognitive processes, including information integration, synaptic plasticity, and inter-regional communication (4–8). Gamma oscillations are known to interact with lower frequency oscillations (e.g., theta, alpha) through cross-frequency coupling (CFC), a mechanism thought to facilitate the coordination of distributed neural networks (9). In the cognitive biotype of depression, abnormalities in gamma oscillations and their coupling with low-frequency rhythms may underlie the observed cognitive deficits, though the precise mechanisms remain poorly understood. Emerging evidence suggests that gamma-band abnormalities in depression are not isolated phenomena but are closely linked to alterations in low-frequency oscillations. For instance, impaired theta-gamma coupling has been associated with working memory deficits in depressed individuals Moreover, hippocampal theta-gamma phase-amplitude coupling integrates cognitive control and working memory storage across brain areas, thereby suggesting a potential mechanism for top-down control over sensory-driven processes (10, 11). Similarly, disrupted low-frequency coupling with high-gamma frequency has been observed in patients with attentional impairments in children with Attention deficit and hyperactivity disorder (12). These findings highlight the potential role of cross-frequency interactions in the cognitive pathology of depression.

Despite these advances, critical gaps remain in our understanding of CFC in the cognitive biotype of depression. First, it is unclear whether specific patterns of low frequency-gamma coupling are associated with this biotype. Second, the functional significance of altered CFC in relation to specific cognitive domains (e.g., executive function, memory) has not been systematically investigated.

Therefore, this study aims to address these gaps by investigating cross-frequency coupling between low-frequency and gamma oscillations (delta, theta, alpha, beta) in individuals with the cognitive biotype of depression. Using high-density electroencephalography (EEG) and advanced signal processing techniques, we will characterize CFC patterns during resting state and cognitive task performance. We hypothesize that the cognitive biotype of depression is associated with distinct patterns of low frequency-gamma coupling, which correlate with specific cognitive deficits and may serve as potential targets for neuromodulation therapies.

## 2 Methods and materials

### 2.1 Participants

This study recruited 141 participants aged 12-60 years diagnosed with unipolar depression without psychotic symptoms, as per DSM-5 criteria, from Beijing Anding Hospital. To minimize the influence of transient emotional states, we chose depressed patients in remission, inclusion criteria required a Visual Analogue Scale score of 5 or higher in emotion. Exclusion criteria comprise comorbid obsessive-compulsive disorder, substance use disorders, or inability to complete study assessments. Following screening and subgroup classification, the final cohort for analysis consisted of 56 individuals classified as the cognitive impairment (CI) subgroup and 85 as the non-cognitive impairment (NCI) subgroup. All participants provided written informed consent, and the study protocol was approved by the Ethics Committee of Beijing Anding Hospital.

### 2.2 Cognitive evaluation by MCCB

Cognitive function in depressed patients was assessed using the MATRICS Consensus Cognitive Battery (MCCB), a comprehensive neuropsychological test battery comprising seven cognitive domains: attention/vigilance, working memory, speed of processing, verbal learning, visual learning, reasoning/problem solving, and social cognition (13). Raw scores were converted to age-, sex-, and education-adjusted T-scores using standardized normative data (14). Participants were classified into the cognitive impairment (CI) biotype if they demonstrated deficits (T-score < 40) in two or more cognitive domains, based on established clinical criteria for defining cognitive impairment in depression (15). Participants completed the MCCB cognitive assessment and then received the resting-state EEG recording.

### 2.3 Scalp encephalographic data acquisition

Neural oscillation data were acquired using a 24-channel wireless EEG system (DSI-24; Wearable Sensing, San Diego, CA) configured according to the international 10-20 electrode placement system. All recordings were conducted in an electromagnetically shielded chamber under controlled low-light conditions. During resting-state EEG acquisition, participants were instructed to maintain a calm, relaxed, and alert state while seated comfortably. EEG data collected for approximately 5 minutes under two conditions: eyes-opened and eyes-closed. Signals from 19 active channels were recorded, with FPz serving as the reference electrode and the left earlobe as the ground, while Cz was used as the primary recording site. Continuous EEG data were digitized at a sampling rate of 300 Hz.

### 2.4 Cross-frequency coupling analysis of neural oscillations

Neural oscillation data were processed and analyzed using EEGLAB (https://sccn.ucsd.edu/eeglab/index.php) (16), a MATLAB-based toolbox (The MathWorks, Inc., Natick, MA). Raw EEG signals, acquired at a sampling rate of 300 Hz, were preprocessed using the following pipeline: (1) band-pass filtering (1-90 Hz) with a notch filter at 50 Hz to remove line noise; (2) segmentation into 2-second epochs; (3) identification and removal of artifact-contaminated epochs; and (4) spherical spline interpolation for bad channels. Independent component analysis (ICA) was applied to decompose the neural signals, followed by manual inspection and removal of components associated with artifacts. After ICA cleaning and baseline correction, the data were re-referenced to the average of all channels. Epochs exceeding the amplitude threshold of ±100 μV were excluded from further analysis.

Phase-amplitude coupling (PAC) between low-frequency and gamma phase was assessed using preprocessed continuous EEG data. After preprocessing is completed, we will concatenate all valid epochs and perform bandpass filtering based on this. Subsequently, we use the Hilbert transform to extract the time series of low-frequency phase and high-frequency amplitude for subsequent phase-amplitude coupling (PAC) analysis (17). Low-frequency phase and high-frequency amplitude time series were extracted with a band-pass filter bandwidth of 1 Hz, yielding 29 phase frequency bands and 34 amplitude frequency bands. The modulation index (MI) was calculated for all frequency pairs across channels using the Kullback-Leibler divergence method. To account for potential spurious coupling, z-scores of MIs were computed using a surrogate data approach. The PAC values were obtained by averaging MIs across the low-frequency phase frequency and gamma amplitude frequency ranges.

### 2.5 Statistical analysis

Statistical analyses were performed using SPSS (version 28.0; IBM Corp., Armonk, NY). Continuous variables are presented as mean ± standard deviation (SD). Group comparisons of cross-frequency coupling measures between the cognitive impairment (CI) and non-cognitive impairment (NCI) subgroups were conducted using independent-sample t-tests, with false discovery rate (FDR) correction for multiple comparisons. Pearson correlation analysis was utilized to explore the relationship of the correlation of cross-frequency coupling with cognitive function in depression at eyes-closed state. The significance threshold was set at *P < 0.05, **P < 0.01, and ***P < 0.001, with all tests being two-tailed.

## 3 Results

### 3.1 Demographic and clinical characteristics of the cognitive biotype

We divided depressed patients into cognitive biotype and non-cognitive impairment subgroup and then compared their demographic and clinical characteristics. These characteristics of the subjects are presented in **Table 1**. The chi-square test revealed that the sex distribution of the CI and NCI subgroup were not statistically significant (*p* > 0.05). While the Mann-Whitney U test showed that patients of the CI group were younger (Z =4.488, *p* < 0.001), had fewer education years (Z = 3.551, *p* < 0.001), and had lower age of onset (Z =4.773, *p* < 0.001). There was no significant difference in the number of episodes and duration of illness between the two groups. These results suggest that younger patients with depression were more likely to develop cognitive impairment.

**Table 1:** Demographic and clinical characteristics of cognitive biotype in MDD.

Characteristics	CI	NCI	Total	*X^2^/Z*	*P*
Number of subjects	N=56	N=85	N=141	—	—
Sex (male/female)	21/35	27/58	48/93	0.495	0.482^a^
Age (years)	26.64 ± 10.05	35.78 ± 11.29	32.15 ± 11.67	4.488	0.000^b^
Education (years)	13.52 ± 3.76	15.88 ± 2.71	14.94 ± 3.36	3.551	0.000^b^
Age of onset (years)	20.57 ± 9.10	29.31 ± 10.89	25.57 ± 11.06	4.773	0.000^b^
Number of episodes	2.54 ± 2.49	2.05 ± 1.62	2.26 ± 2.04	-0.245	0.806^b^
Duration of illness (months)	6.15 ± 6.09	5.91 ± 5.52	6.01 ± 5.75	0.129	0.898^b^
VAS	6.12 ± 1.33	6.56 ± 0.85	6.38 ± 1.09	3.032	0.002 ^b^

^a^chi-square test; ^b^Mann-Whitney U test.

### 3.2 Cross-frequency coupling between low frequency and low gamma at eyes-closed state decreased in cognitive biotype of depression

**Figure 1:**
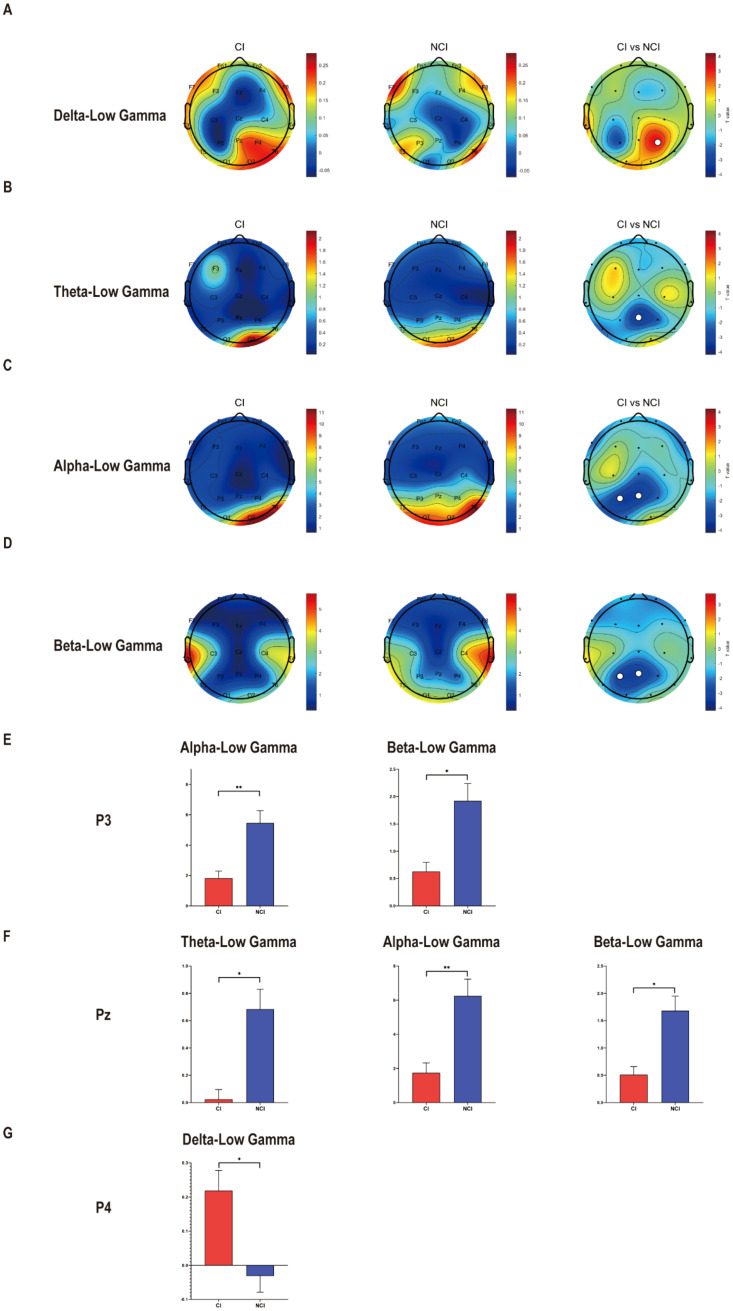
Cross-frequency coupling decreased between low frequency with low gamma at eyes-closed state in cognitive biotype of depression. **(A)** Cross-frequency coupling between delta with gamma in P4 increased in cognitive biotype. **(B)** Cross-frequency coupling between theta with gamma in Pz decreased in cognitive biotype. **(C)** Cross-frequency coupling between alpha with gamma in P3 and Pz decreased in cognitive biotype. **(D)** Cross-frequency coupling between beta with gamma in P3 and Pz decreased in cognitive biotype. **(E)** In P3, cross-frequency coupling between alpha, beta with gamma decreased in cognitive biotype. **(F)** In Pz, cross-frequency coupling between theta, alpha, beta with gamma decreased in cognitive biotype. **(G)** In P4, cross-frequency coupling between delta with gamma increased in cognitive biotype. CI, cognitive impairment group, NCI, non-cognitive impairment. ○ represents channels with statistically significant differences. *P < 0.05, **P < 0.01.

To identify the cross-frequency coupling between low frequency and gamma in cognitive biotype of depression, we calculated the PAC values of neural oscillations. Interestingly, we found that PAC values decreased in cognitive biotype. Specifically, cross-frequency coupling between theta (Pz: *t* =-3.512, FDR-corrected p = 0.011), alpha (P3: *t* =-3.377, FDR-corrected p = 0.009; Pz: *t* =-3.451, FDR-corrected p = 0.009), beta (P3: *t* =-3.129, FDR-corrected p = 0.020; Pz: *t* =-3.333, FDR-corrected p = 0.020) with low gamma decreased in cognitive biotype. However, cross-frequency coupling between delta with low gamma increased in cognitive biotype (P4: *t* = 3.314, FDR-corrected p = 0.022) (**Figure 1**). While other cross-frequency coupling or channels exhibited no significant differences in two subgroups (FDR-corrected p > 0.05). These results indicated that the cross-frequency coupling between low frequency and gamma occurred in the parietal lobe in cognitive biotype of depression.

### 3.3 Cross-frequency coupling between low frequency and gamma at eyes-opened state remained unchanged in cognitive biotype of depression

**Figure 2:**
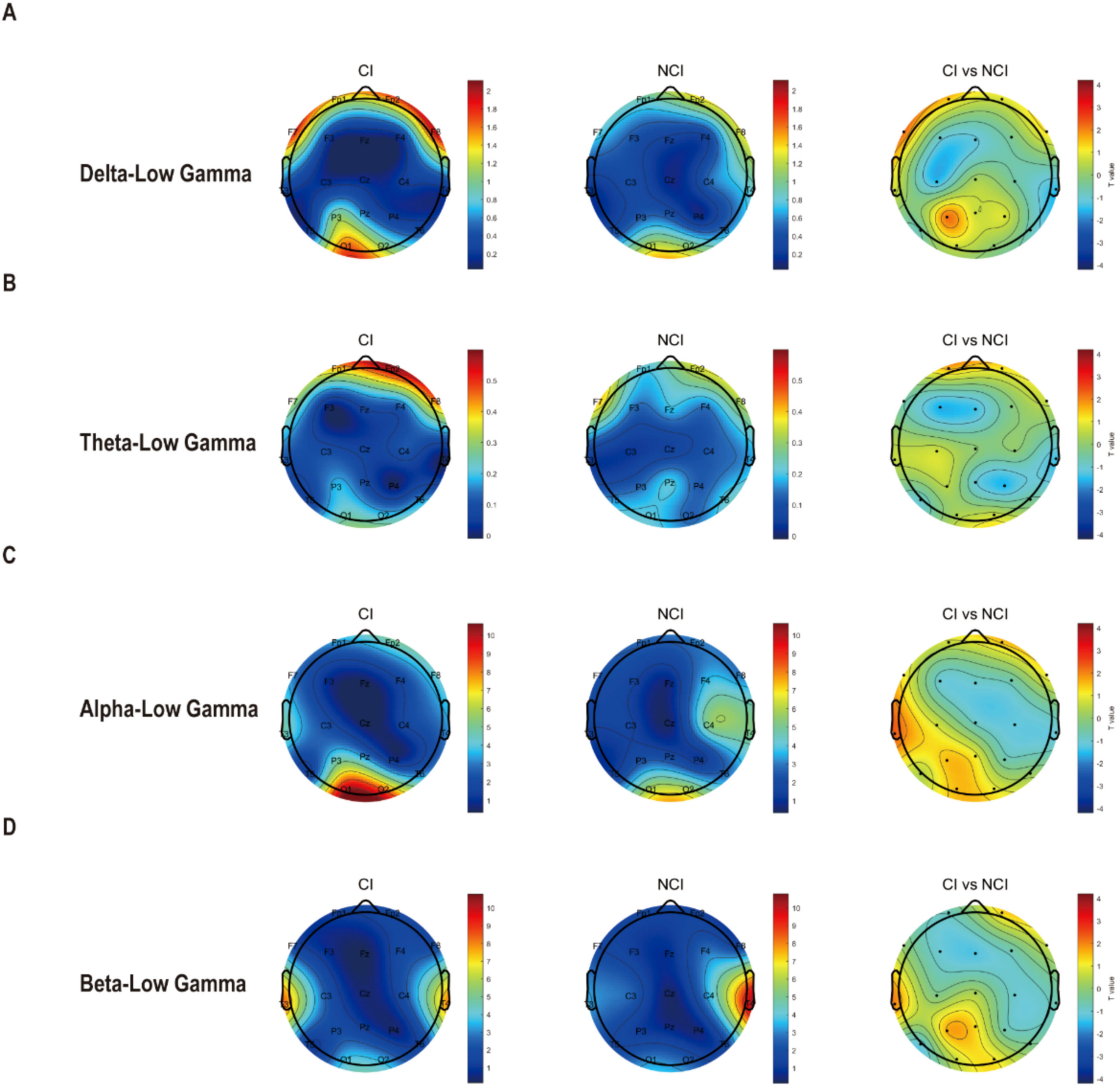
Cross-frequency coupling between low frequency with gamma existed no difference at eyes-opened state in cognitive biotype of depression. **(A)** Cross-frequency coupling between delta with low-gamma existed no difference in cognitive biotype. **(B)** Cross-frequency coupling between theta with low-gamma existed no difference in cognitive biotype. **(C)** Cross-frequency coupling between alpha with low-gamma existed no difference in cognitive biotype. **(D)** Cross-frequency coupling between beta with low-gamma existed no difference in cognitive biotype. CI, cognitive impairment group; NCI, non-cognitive impairment.

Furthermore, we also analyzed the cross-frequency coupling between low frequency and gamma at eyes-opened state. Unfortunately, we found that cross-frequency coupling between delta, theta, alpha, beta with gamma remained unchanged in cognitive biotype. But interestingly, PAC values of theta-alpha (C3: *t* =3.087, FDR-corrected p = 0.046) decreased in cognitive biotype (**Figure 2**). While other cross-frequency coupling or channels exhibited no significant differences in two subgroups (FDR-corrected p > 0.05). These results indicated that the cross-frequency coupling between theta and alpha occurred in the left sensorimotorcortex in cognitive biotype of depression.

### 3.4 The correlation of cross-frequency coupling with cognitive function in depression at eyes-closed state

**Figure 3:**
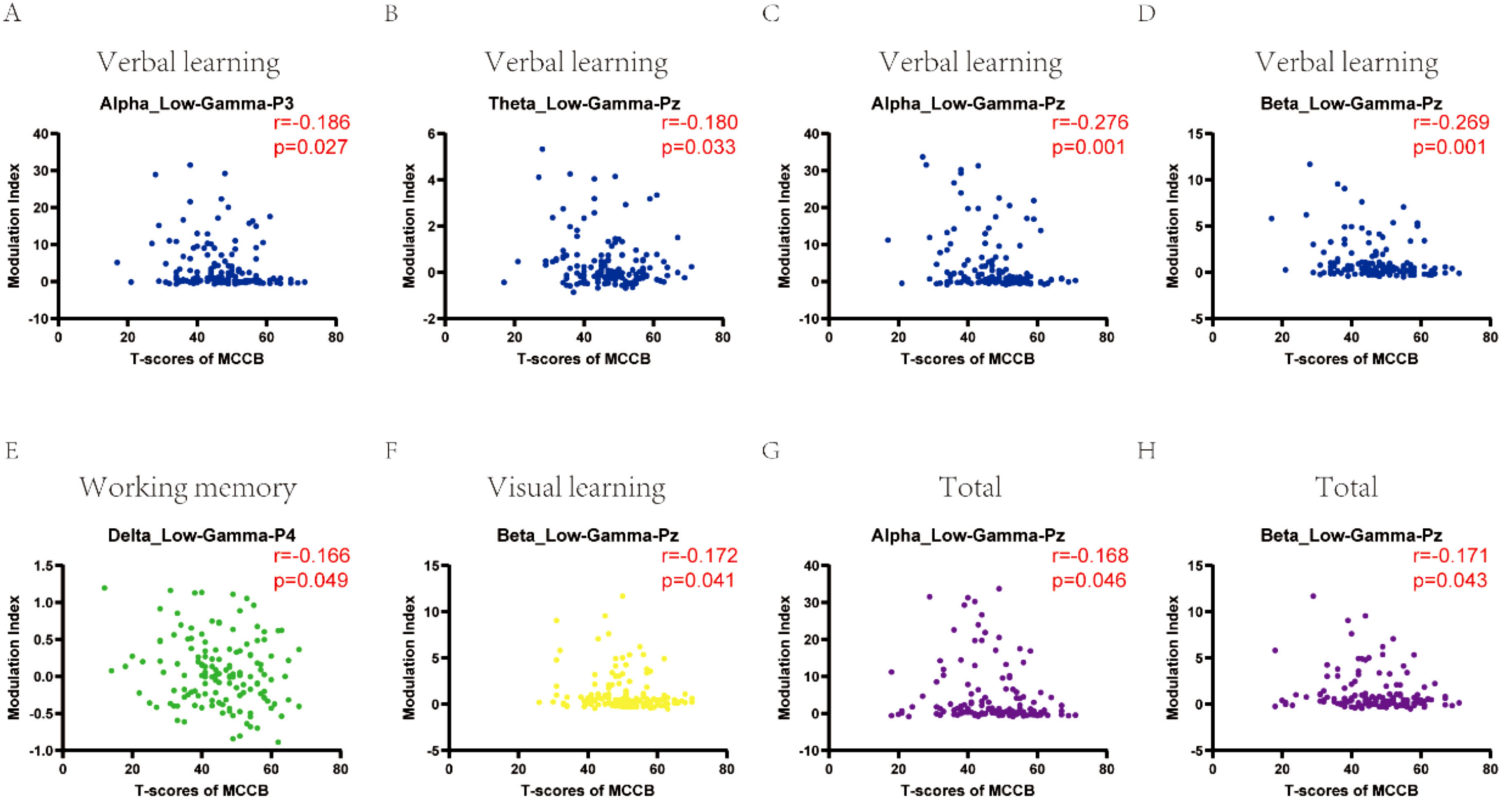
The correlation of cross-frequency coupling with cognitive function in depression at eyes-closed state. **(A-D)** Verbal learning was correlated with cross-frequency coupling between alpha and low-gamma in P3, Pz channels; theta and low-gamma in Pz channel; beta and low-gamma in Pz channel. **(E)** Working memory correlated with cross-frequency coupling between delta and low-gamma in P4 channel. **(F)** Visual learning was correlated with cross-frequency coupling between beta and low-gamma in Pz channel. **(G, H)** Total scores of MCCB showed significant correlation with cross-frequency coupling between alpha and low-gamma, beta and low-gamma in Pz channel. CI, cognitive impairment group; NCI, non-cognitive impairment.

To further explore the relationship between cross-frequency coupling and cognitive function in depression, we did correlation analysis. Significant correlations between cognitive function and cross-frequency coupling were observed (**Figure 3**). Verbal learning was negatively correlated with cross-frequency coupling between alpha and low-gamma in P3 channel (*r* = -0.186, P = 0.027), Pz channel (*r* = -0.276, P = 0.001);theta and low-gamma in Pz channel (*r* = -0.180, P = 0.033);beta and low-gamma in Pz channel (*r* = -0.269, P = 0.001). Working memory was negatively correlated with cross-frequency coupling between delta and low-gamma in P4 channel (*r* = -0.166, P = 0.049). Visual learning was negatively correlated with cross-frequency coupling between beta and low-gamma in Pz channel (*r* = -0.172, P = 0.041). While total scores of MCCB showed significant negative correlation with cross-frequency coupling between alpha and low-gamma in Pz channel (*r* = -0.168, P = 0.046), beta and low-gamma in Pz channel (*r* = -0.171, P = 0.043).

## 4 Discussion

Here, we found that cross-frequency coupling decreased in cognitive biotype of depression. Specifically, cross-frequency coupling between theta, alpha, and beta with low gamma decreased at eyes-closed state in cognitive biotype. While cross-frequency coupling exhibited no significant differences at eyes-opened state in two subgroups. Furthermore, significant correlations between cognitive function and cross-frequency coupling were observed. Our study identifies state- and domain-specific disruptions in cross-frequency coupling within a cognitive biotype of major depressive disorder, linking aberrant neural oscillatory coordination to cognitive dysfunction. These findings deepen our understanding of the neurophysiological substrates of cognitive impairment in depression and highlight potential biomarkers for targeted interventions.

Cross-frequency coupling is a fundamental mechanism in the brain that facilitates communication between different neural oscillations, playing a crucial role in cognitive processes. The eyes-opened (EO) state may be interfered by eye movement and visual processing due to potentially more noises in eye-opened conditions. The eyes-closed (EC) state engages the default mode network (DMN), which is hyperactive in depression and associated with rumination (18). Diminished CFC may reflect a failure of slow oscillations to temporally organize local gamma activity, disrupting top-down control and inter-network integration. Conversely, the normalization of CFC differences during the eyes-opened state suggests that external sensory inputs recruit compensatory visual and attentional networks, transiently overriding intrinsic coupling deficits. This parallels findings in schizophrenia, where sensory-driven tasks partially restore oscillatory dynamics (19), and underscores the state-dependent nature of neural dysregulation in psychiatric disorders. Research has shown that CFC is involved in various cognitive tasks and processes. For instance, the synchronization between theta and gamma rhythms in the hippocampus is crucial for memory processes, and disruptions in this coupling can impair memory function (20). Similarly, the coupling between alpha and theta rhythms has been associated with cognitive performance, with harmonic relationships between these frequencies facilitating efficient cognitive processing (21). In the context of attention, age-related changes in EEG coupling, particularly in theta and alpha bands, have been observed, indicating that these couplings are vital for maintaining cognitive performance across the lifespan (22). In pathological conditions, such as schizophrenia, increased theta-phase gamma-amplitude coupling has been reported, which may reflect compensatory mechanisms in response to dysfunctional neural networks (23). Conversely, in conditions like Alzheimer’s disease, alterations in brain connectivity, including changes in CFC, have been associated with cognitive decline, highlighting the importance of CFC in maintaining cognitive health (24).

Moreover, studies have demonstrated that CFC patterns can vary depending on task demands and cognitive states. For example, during visual working memory tasks, enhanced phase-amplitude coupling between theta/alpha phases and beta amplitudes in the temporal cortex has been linked to memory maintenance, suggesting that CFC supports the coordination of brain regions involved in memory (25). In the auditory cortex, distinct CFC patterns have been observed for top-down and bottom-up processing, indicating that different types of information processing rely on specific CFC configurations (26).

We reported a kind of state-dependent CFC abnormalities. The reduced theta, alpha, and beta coupling with gamma oscillations during the eyes-closed (EC) state suggests impaired hierarchical neural integration in the cognitive biotype. Diminished CFC may reflect a failure of low-frequency rhythms to temporally organize local high-frequency activity, disrupting information transfer between large-scale networks (9). Notably, the absence of group differences during the eyes-opened (EO) state implies that external sensory inputs recruit compensatory mechanisms (e.g., visual and dorsal attention networks), transiently masking intrinsic coupling deficits. This aligns with studies showing state-dependent DMN dysregulation in schizophrenia and other cognitive disorders (19). Overall, the decrease in CFC between other frequency bands and gamma in cognitive biotypes may indicate a potential disruption in the context of resting-state neural dynamics. This reduction in coupling could lead to impairments in cognitive functions such as memory, attention, and information processing, emphasizing the need for further research to understand the implications of these changes and develop interventions to mitigate their effects.

Also, we found cognitive domain-specific coupling patterns. Correlations between alpha-low gamma CFC over parietal channels (P3, Pz) and verbal learning performance suggest that alpha oscillations gate attention to semantically relevant information, while gamma synchrony binds features into coherent memory traces (27). Reduced alpha-gamma coupling may weaken top-down control during encoding, impairing the integration of verbal information. The association of delta-low gamma CFC at P4 with working memory supports the “temporal scaffolding” hypothesis, where delta oscillations provide a slow temporal framework to sequence gamma-mediated item representations (28). Dysregulated delta-gamma coupling could fragment the temporal structure of working memory maintenance. Beta-low gamma CFC at Pz correlated with visual learning, consistent with beta’s role in maintaining persistent cortical states for post-perceptual processing (29). Beta-gamma interactions may stabilize visual object representations during consolidation, with disruptions leading to inefficient learning. The strong linkage between MCCB scores and alpha/beta-low gamma CFC at Pz underscores the parietal cortex’s role as a hub for integrating multisensory information. Parietal alpha and beta rhythms may regulate the balance between DMN-mediated internal processing and task-oriented attention, with CFC reductions reflecting a collapse of this dynamic equilibrium (30).

CFC abnormalities may arise from GABAergic dysfunction, particularly in parvalbumin-positive interneurons that generate gamma oscillations and synchronize them to slower rhythms (31). Animal models show that GABA hypofunction disrupts gamma coherence and cross-frequency coordination, impairing cognitive performance (32). Pharmacological agents targeting GABA-glutamate balance (e.g., NMDA modulators) or neuromodulation techniques (e.g., transcranial alternating current stimulation) could restore CFC dynamics (33).

While our EEG-based findings provide spatial resolution constraints, future studies should integrate high-density EEG, MEG, or intracranial recordings to localize CFC sources (34). Longitudinal designs are needed to determine whether CFC alterations predict cognitive decline. Additionally, task-based paradigms could clarify how CFC dynamics adapt during active cognitive processing. Acknowledge EEG’s spatial resolution limitations but argue for its utility in capturing dynamic CFC properties.

Our findings demonstrate state- and domain-specific alterations in cross-frequency coupling within a cognitive biotype, linking disrupted neural oscillatory coordination to cognitive impairment. These results advance the understanding of neurophysiological mechanisms underlying cognitive deficits and highlight potential biomarkers for precision depression. CFC metrics could stratify depression into cognitive vs. affective biotypes, guiding personalized treatment. For instance, patients with alpha-gamma decoupling may benefit from alpha-frequency transcranial magnetic stimulation (TMS) to enhance top-down control. Those with delta-gamma deficits might respond to slow-wave synchronized cognitive remediation. Real-time CFC neurofeedback could train patients to self-modulate aberrant coupling patterns.

## Data Availability

The original contributions presented in the study are included in the article/supplementary material. Further inquiries can be directed to the corresponding author.

## References

[1] Drysdale AT Grosenick L Downar J Dunlop K Mansouri F Meng Y. Resting-state connectivity biomarkers define neurophysiological subtypes of depression. Nat Med. (2017) 23:28–38. doi: 10.1038/nm.4246 27918562 PMC5624035

[2] Tozzi L Zhang X Pines A Olmsted AM Zhai ES Anene ET. Personalized brain circuit scores identify clinically distinct biotypes in depression and anxiety. Nat Med. (2024) 30:2076–87. doi: 10.1038/s41591-024-03057-9 PMC11271415 38886626

[3] Hack LM Tozzi L Zenteno S Olmsted AM Hilton R Jubeir J. A cognitive biotype of depression and symptoms, behavior measures, neural circuits, and differential treatment outcomes: A prespecified secondary analysis of a randomized clinical trial. JAMA Netw Open. (2023) 6:e2318411. doi: 10.1001/jamanetworkopen.2023.18411 37318808 PMC10273022

[4] Adaikkan C Tsai L-H. Gamma entrainment: impact on neurocircuits, glia, and therapeutic opportunities. Trends Neurosci. (2020) 43:24–41. doi: 10.1016/j.tins.2019.11.001 31836315

[5] Fitzgerald PJ Watson BO. Gamma oscillations as a biomarker for major depression: an emerging topic. Transl Psychiatry. (2018) 8:177. doi: 10.1038/s41398-018-0239-y 30181587 PMC6123432

[6] Fries P. Rhythms for cognition: communication through coherence. Neuron. (2015) 88:220–35. doi: 10.1016/j.neuron.2015.09.034 PMC4605134 26447583

[7] Lundqvist M Rose J Herman P Brincat SL Buschman TJ Miller EK. Gamma and beta bursts underlie working memory. Neuron. (2016) 90:152–64. doi: 10.1016/j.neuron.2016.02.028 PMC5220584 26996084

[8] Miljevic A Bailey NW Murphy OW Perera MPN Fitzgerald PB. Alterations in EEG functional connectivity in individuals with depression: A systematic review. J Affect Disord. (2023) 328:287–302. doi: 10.1016/j.jad.2023.01.126 36801418

[9] Canolty RT Knight RT. The functional role of cross-frequency coupling. Trends Cognit Sci. (2010) 14:506–15. doi: 10.1016/j.tics.2010.09.001 PMC3359652 20932795

[10] Daume J Kamiński J Schjetnan AGP Salimpour Y Khan U Kyzar M. Control of working memory by phase-amplitude coupling of human hippocampal neurons. Nature. (2024) 629:393–401. doi: 10.1038/s41586-024-07309-z 38632400 PMC11078732

[11] Papaioannou O Crespo LP Clark K Ogbuagu NN Alliende LM Silverstein SM. Is cortical theta-gamma phase-amplitude coupling memory-specific? Brain Sci. (2022) 12(9):1131. doi: 10.3390/brainsci12091131 36138867 PMC9496728

[12] Liu X Sun L Zhang D Wang S Hu S Fang B. Phase-amplitude coupling brain networks in children with attention-deficit/hyperactivity disorder. Clin EEG Neurosci. (2022) 53:399–405. doi: 10.1177/15500594221086195 35257602

[13] Shi C Kang L Yao S Ma Y Li T Liang Y. The MATRICS consensus cognitive battery (MCCB): co-norming and standardization in China. Schizophr Res. (2015) 169:109–15. doi: 10.1016/j.schres.2015.09.003 PMC4916953 26441005

[14] Shi C Kang L Yao S Ma Y Li T Liang Y. What is the optimal neuropsychological test battery for schizophrenia in China? Schizophr Res. (2019) 208:317–23. doi: 10.1016/j.schres.2019.01.034 PMC6544499 30718121

[15] Lai S Zhong S Wang Y Zhang Y Xue Y Zhao H. The prevalence and characteristics of MCCB cognitive impairment in unmedicated patients with bipolar II depression and major depressive disorder. J Affect Disord. (2022) 310:369–76. doi: 10.1016/j.jad.2022.04.153 35504401

[16] Delorme A Makeig S. EEGLAB: an open source toolbox for analysis of single-trial EEG dynamics including independent component analysis. J Neurosci Methods. (2004) 134:9–21. doi: 10.1016/j.jneumeth.2003.10.009 15102499

[17] Tort AB Komorowski R Eichenbaum H Kopell N. Measuring phase-amplitude coupling between neuronal oscillations of different frequencies. J Neurophysiol. (2010) 104:1195–210. doi: 10.1152/jn.00106.2010 PMC2941206 20463205

[18] Hamilton JP Farmer M Fogelman P Gotlib IH. Depressive rumination, the default-mode network, and the dark matter of clinical neuroscience. Biol Psychiatry. (2015) 78:224–30. doi: 10.1016/j.biopsych.2015.02.020 PMC4524294 25861700

[19] Uhlhaas PJ Singer W. Neuronal dynamics and neuropsychiatric disorders: toward a translational paradigm for dysfunctional large-scale networks. Neuron. (2012) 75:963–80. doi: 10.1016/j.neuron.2012.09.004 22998866

[20] Xu X Zheng C Zhang T. Reduction in LFP cross-frequency coupling between theta and gamma rhythms associated with impaired STP and LTP in a rat model of brain ischemia. Front Comput Neurosci. (2013) 7:27. doi: 10.3389/fncom.2013.00027 23576981 PMC3617395

[21] Rodriguez-Larios J Alaerts K. Tracking transient changes in the neural frequency architecture: harmonic relationships between theta and alpha peaks facilitate cognitive performance. J Neurosci. (2019) 39:6291–8. doi: 10.1523/jneurosci.2919-18.2019 PMC6687903 31175211

[22] Li L Zhao D. Age-related inter-region EEG coupling changes during the control of bottom-up and top-down attention. Front Aging Neurosci. (2015) 7:223. doi: 10.3389/fnagi.2015.00223 26648868 PMC4664751

[23] Won GH Kim JW Choi TY Lee YS Min KJ Seol KH. Theta-phase gamma-amplitude coupling as a neurophysiological marker in neuroleptic-naïve schizophrenia. Psychiatry Res. (2018) 260:406–11. doi: 10.1016/j.psychres.2017.12.021 29253805

[24] Vecchio F Miraglia F Piludu F Granata G Romanello R Caulo M. Small World” architecture in brain connectivity and hippocampal volume in Alzheimer’s disease: a study via graph theory from EEG data. Brain Imaging Behav. (2017) 11:473–85. doi: 10.1007/s11682-016-9528-3 26960946

[25] Daume J Gruber T Engel AK Friese U. Phase-amplitude coupling and long-range phase synchronization reveal frontotemporal interactions during visual working memory. J Neurosci. (2017) 37:313–22. doi: 10.1523/jneurosci.2130-16.2016 PMC6596581 28077711

[26] Márton CD Fukushima M Camalier CR Schultz SR Averbeck BB. Signature patterns for top-down and bottom-up information processing via cross-frequency coupling in macaque auditory cortex. eNeuro. (2019) 6(2):ENEURO.0467-18. doi: 10.1523/eneuro.0467-18.2019 PMC6520641 31088914

[27] Jensen O Mazaheri A. Shaping functional architecture by oscillatory alpha activity: gating by inhibition. Front Hum Neurosci. (2010) 4:186. doi: 10.3389/fnhum.2010.00186 21119777 PMC2990626

[28] Lisman JE Jensen O. The θ-γ neural code. Neuron. (2013) 77:1002–16. doi: 10.1016/j.neuron.2013.03.007 PMC3648857 23522038

[29] Engel AK Fries P. Beta-band oscillations–signalling the status quo? Curr Opin Neurobiol. (2010) 20:156–65. doi: 10.1016/j.conb.2010.02.015 20359884

[30] Corbetta M Shulman GL. Control of goal-directed and stimulus-driven attention in the brain. Nat Rev Neurosci. (2002) 3:201–15. doi: 10.1038/nrn755 11994752

[31] Sohal VS Zhang F Yizhar O Deisseroth K. Parvalbumin neurons and gamma rhythms enhance cortical circuit performance. Nature. (2009) 459:698–702. doi: 10.1038/nature07991 19396159 PMC3969859

[32] Gonzalez-Burgos G Lewis DA. GABA neurons and the mechanisms of network oscillations: implications for understanding cortical dysfunction in schizophrenia. Schizophr Bull. (2008) 34:944–61. doi: 10.1093/schbul/sbn070 PMC2518635 18586694

[33] Herrmann CS Strüber D Helfrich RF Engel AK. EEG oscillations: From correlation to causality. Int J Psychophysiol. (2016) 103:12–21. doi: 10.1016/j.ijpsycho.2015.02.003 25659527

[34] Hipp JF Engel AK Siegel M. Oscillatory synchronization in large-scale cortical networks predicts perception. Neuron. (2011) 69:387–96. doi: 10.1016/j.neuron.2010.12.027 21262474

